# Long-Term Monitoring of Dolphin Biosonar Activity in Deep Pelagic Waters of the Mediterranean Sea

**DOI:** 10.1038/s41598-017-04608-6

**Published:** 2017-06-28

**Authors:** Francesco Caruso, Giuseppe Alonge, Giorgio Bellia, Emilio De Domenico, Rosario Grammauta, Giuseppina Larosa, Salvatore Mazzola, Giorgio Riccobene, Gianni Pavan, Elena Papale, Carmelo Pellegrino, Sara Pulvirenti, Virginia Sciacca, Francesco Simeone, Fabrizio Speziale, Salvatore Viola, Giuseppa Buscaino

**Affiliations:** 1 Bioacoustics Lab, IAMC Capo Granitola, National Research Council, Torretta Granitola (TP), Italy; 2 ENEA - Observations and Analyses of Earth and Climate, Palermo, Italy; 3 Dipartimento di Fisica ed Astronomia, University of Catania, Catania, Italy; 4 Istituto Nazionale di Fisica Nucleare (INFN) - Laboratori Nazionali del Sud, Catania, Italy; 5 Dip. Scienze Chimiche, Biologiche, Farmaceutiche ed Ambientali, University of Messina, Messina, Italy; 6 Centro Interdisciplinare di Bioacustica e Ricerche Ambientali (CIBRA), Dipartimento di Scienze della Terra e dell’Ambiente, University of Pavia, Pavia, Italy; 7 Istituto Nazionale di Fisica Nucleare (INFN), Sezione di Bologna, Bologna, Italy; 8 Istituto Nazionale di Fisica Nucleare (INFN), Sezione di Roma1, Roma, Italy

## Abstract

Dolphins emit short ultrasonic pulses (clicks) to acquire information about the surrounding environment, prey and habitat features. We investigated *Delphinidae* activity over multiple temporal scales through the detection of their echolocation clicks, using long-term Passive Acoustic Monitoring (PAM). The Istituto Nazionale di Fisica Nucleare operates multidisciplinary seafloor observatories in a deep area of the Central Mediterranean Sea. The Ocean noise Detection Experiment collected data offshore the Gulf of Catania from January 2005 to November 2006, allowing the study of temporal patterns of dolphin activity in this deep pelagic zone for the first time. Nearly 5,500 five-minute recordings acquired over two years were examined using spectrogram analysis and through development and testing of an automatic detection algorithm. Echolocation activity of dolphins was mostly confined to nighttime and crepuscular hours, in contrast with communicative signals (whistles). Seasonal variation, with a peak number of clicks in August, was also evident, but no effect of lunar cycle was observed. Temporal trends in echolocation corresponded to environmental and trophic variability known in the deep pelagic waters of the Ionian Sea. Long-term PAM and the continued development of automatic analysis techniques are essential to advancing the study of pelagic marine mammal distribution and behaviour patterns.

## Introduction

Studying marine mammal ecology and behaviour in the vast ocean environment, particularly in remote non-coastal areas, presents a significant challenge^1^. Classic visual and photo-identification surveys, provide information only on the surface activities of these highly mobile animals and only for limited time periods. Passive Acoustic Monitoring (PAM) offers a non-invasive and reliable method to survey acoustically active animals and provide information on their distributions and activities at high spatiotemporal resolutions^2^. Technological innovations have allowed the development of new data acquisition systems to obtain long-term and dynamic information on sound producing marine organisms. Among the marine organisms that emit sounds, dolphins fill an important ecological niche as top predators in pelagic ecosystems, and have exceptional acoustic capabilities^3, 4^. Sound production and reception is critical for delphinids to navigate, find food, coordinate with others, and detect predators. By recording and studying their sounds, insight into their ecological and trophic dynamics can be obtained. Signal identification and processing of acoustic datasets are essential tools needed to advance our ability to study dolphins in the wild^2^, especially in inaccessible and understudied populations. The development of algorithms to analyse large datasets are increasingly needed, as they present many advantages in working time, statistical analysis, and checks on the results compared to conventional manual methods.

In particular, long-term acoustic monitoring allows, in contrast with short-term daily surveys, the study of circadian, monthly, and seasonal dynamics. Life in marine ecosystems is governed by a multitude of environmental cycles, not only at daily scales, but also by cycles with shorter and longer periods such as tides, lunar phases, and seasons^5^. Marine organisms have adapted to these cycles over millions of years, and biological rhythms and ecological dynamics are commonly synchronized with environmental periodicities. External stimuli – such as light – often affect the timing of these periodic phenomena^5^. Given these relationships between marine organism activity and environmental variability over multiple temporal scales, it is not surprising that recent efforts to characterize soundscapes using long-term acoustic monitoring with high temporal resolution have found higher complexity and patterns of fish and invertebrate sound production than had previously been detected by short acoustic surveys^6–9^. Similarly, recent long-term PAM surveys and measurements of temporal patterns in biosonar emissions have greatly advanced the understanding of species distributions and foraging activity of deep-diving odontocetes^10^.

Species belonging to the cetacean Family *Delphinidae* are extremely vocal mammals and their acoustic behaviour plays a fundamental role in both recognition of the environment and in mediating social interactions^3, 4^. Dolphins produce a wide variety of sounds, which can be classified into two main categories: short-broadband ultrasonic clicks (called echolocation or biosonar) and frequency-modulated narrowband whistles^11^. The ultrasonic clicks are used primarily to acquire sensory information on the composition of the surrounding environment and to locate prey or other targets (e.g., inanimate objects, seabed, etc.)^3, 12–14^. The information is obtained via analysis of echoes generated from ensonified objects by sound reception through a region of the lower jaw and transmission into the ear^3^. Dolphins change the temporal and spectral features of their echolocation signals in relation to distance, size, shape and acoustic properties of the focused target^12, 13, 15, 16^. Conversely, whistle signals are typically frequency modulated and within the human hearing range, and are commonly used for intra-species social communication^4^. For example, specific whistles may act as recognition signals for maintaining contact between individuals^11, 17^. Because the production of these different types of sounds are indicative of distinct activities and life history functions, PAM can provide measurements of dolphin ecology and behavioural patterns that are otherwise impossible to monitor in the natural environment.

In the last decade, several automatic detection algorithms were developed to analyse the echolocation signals emitted by Odontocete species^18–20^. Traditionally, marine mammal vocalizations were characterized via time-consuming manual approaches such as spectrogram visualization and listening. However, acquisition of data continuously and during long-term monitoring has encouraged automatic analyses. In the first stages of development of automatic detection algorithms for a particular area and species complex it is imperative to test and confirm the reliability of the detector with human checks^2^. In analysis of broadband echolocation clicks using acoustic monitoring, it is important to account for variability in waveform amplitude and spectral components of the signal due to the dolphin’s distance and orientation in relation to the receiving sensor (hydrophone) position^18^. Alteration of the click during its propagation through seawater is due to the different attenuation of the sound as a function of the frequency: high frequency components are attenuated more than low frequency ones^21^.

**Figure 1:**
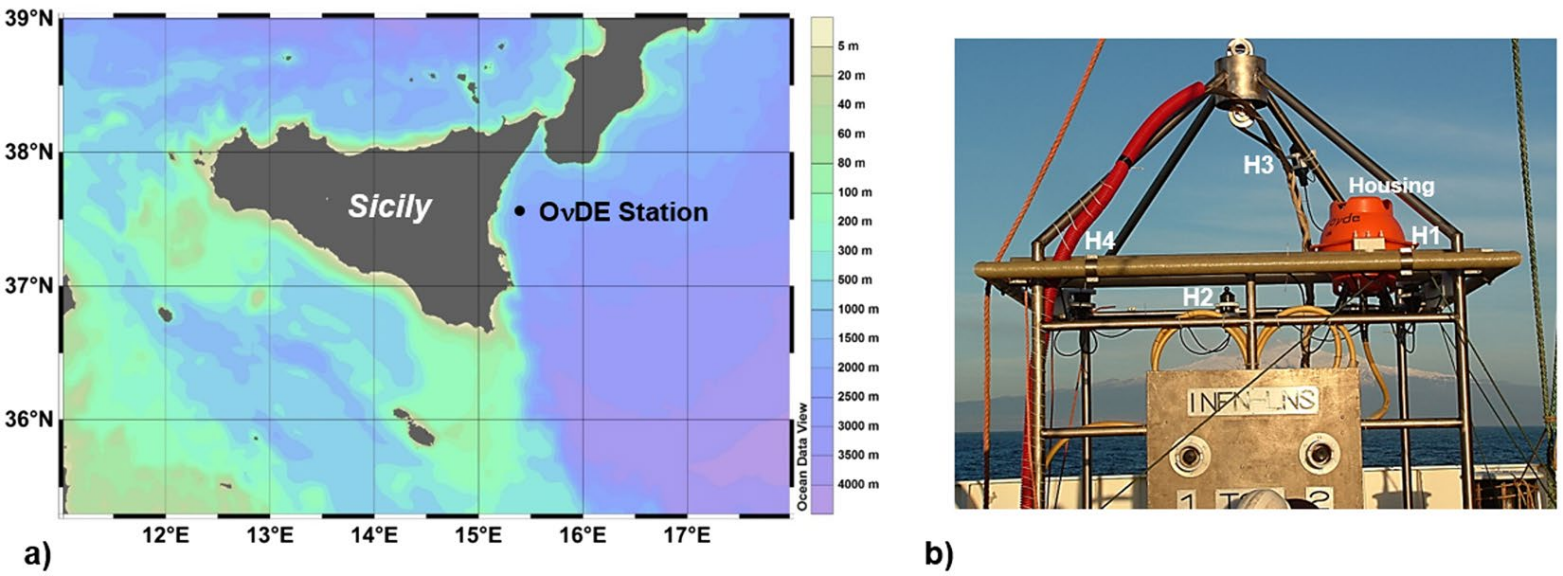
The OνDE station. (**a**) Site of installation of the OνDE acoustic array (NEMO Test Site, 2,100 meters of depth, Western Ionian Sea)^29^. *Ocean Data View* software (Version 4.0, http://odv.awi.de)^49^. (**b**) Photo of the OνDE frame before deployment. Hydrophones and electronics housing are labelled (H1, H2, H3, H4, Housing)^29^. Copyright:© 2015 Caruso *et al*. This is an open access figure distributed under the terms of the Creative Commons Attribution License.

The Istituto Nazionale di Fisica Nucleare (INFN), in collaboration with the Istituto Nazionale di Geofisica e Vulcanologia (INGV) and within the European Multidisciplinary Seafloor and water column Observatory (EMSO) Research Infrastructure^22^, operates deep-sea stations offshore Eastern Sicily (Italy, Southwestern Ionian Sea)^23, 24^. The INFN is strongly involved in the design and construction of the KM3NeT Cherenkov neutrino telescope^25–27^. To test novel technologies for neutrino detection^28–30^, the Laboratori Nazionali del Sud – INFN built a submarine infrastructure in a test site offshore the Gulf of Catania within the activities of the NEMO (Neutrino Mediterranean Observatory) project. At this site, the Ocean noise Detection Experiment (OνDE) was in operation from January 2005 to November 2006, at a depth of 2,100 meters (Fig. 1)^31^. The OνDE was the first cabled deep-sea acoustic station of the Mediterranean Sea and provided results on long-term acoustic monitoring and cetacean biodiversity^31–34^. The project aimed at studying acoustic noise for future experiments on astroparticle physics^35, 36^, and had significant applications in marine bioacoustics^37^.

Five delphinid species are present in the Western Ionian Sea and produce the acoustic signals described above: bottlenose dolphin (*Tursiops truncatus*), striped dolphin (*Stenella coeruleoalba*), short-beaked common dolphin (*Delphinus delphis*), Risso’s dolphin (*Grampus griseus*) and pilot whale (*Globicephala melas*)^38, 39^. Though little is known about their natural population dynamics, these species represent a range of life history characteristics and habitat use patterns, and all are important top predators in the pelagic marine ecosystem, feeding primarily on fish and squid. The Mediterranean subpopulations are included in the IUCN (International Union for the Conservation of Nature and Natural Resources) Red List of Threatened Species as “vulnerable” or “endangered”^38^. Little is currently known about their distribution offshore Eastern Sicily, thus their status is considered “data deficient”^38^.

In this work, we aimed to characterize - for the first time within the Western Ionian Sea - the circadian and monthly dynamics of dolphin acoustic activity in a pelagic area of the Mediterranean Sea, using sensors installed in the deep-sea. Toward that goal, we developed and tested an automatic detection method for dolphin biosonar signals. First, human-operators investigated the presence of dolphin vocalizations (both clicks and whistles) via spectrogram analysis within the OνDE large dataset (5,494 five-minute long files). Then, a MATLAB algorithm was developed to measure the number of clicks detected in each recorded file. This approach allowed us to reduce data analysis time and obtain further information on the biosonar emission rate. Accuracy of click detection by manual (human-operators) and automatic (algorithm) analysis was compared. Acoustic data were explored across multiple temporal scales and the influence of lunar cycle on biosonar activity of dolphins was also investigated. These echolocation acoustic activity patterns were compared to communication signal patterns (whistles), to better understand potential ecological significance of temporal variation. Finally, the Transmission Loss (TL) of a dolphin click was evaluated to estimate the detection range of the acoustic sensor.

## Results

### Biosonar detection

**Figure 2:**
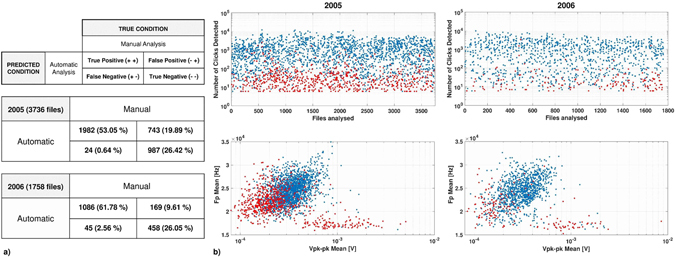
Biosonar detection. (**a**) Confusion matrix shows the comparison between the Manual analysis (*True condition*) and the Automatic analysis (*Predicted condition*). Performance of the algorithm is showed for recordings acquired in 2005 and 2006. In 2005, TP rate (TP/P) was 72%, FP rate (FP/N) was 67%, FN (FN/P) rate was 0.8% and TN (TN/N) rate was 97%. In 2006, they were 86%, 33%, 3.5% and 91% respectively. (**b**) (*top*) Number of clicks recorded per file during 2005 and 2006. (*bottom*) Mean Amplitude (*x-axis*, peak to peak) and mean Frequency Peak (*y-axis*) of the clicks selected in each file. Recordings classified as False Positive are marked in red.

The acoustic presence or absence of dolphin clicks (biosonar) within the OνDE large dataset (5,494 five-minute recordings) was inspected by expert operators through spectrogram visualization (manual analysis). The developed algorithm allowed automatic detection of clicks, marking the number of events recorded per file and collecting information on the selected signals. The algorithm output also allowed a comparison of detection methods using a confusion matrix test. Results of the manual analysis (data binary, presence 1, absence 0) were automatically uploaded for each file. This information considered the instances of *True condition* (columns) and the automatic detection was the *Predicted class* (rows). The recordings were classified in four different categories, in relation to the detection of dolphin clicks by means of the two techniques (True Positive-TP, True Negative-TN, False Positive-FP, and False Negative-FN). The Accuracy ((TP + TN)/TOT) of the automatic detection was 79% for the recordings acquired in 2005 and 87% for 2006 (Fig. 2a).

Results showed good reliability of the algorithm developed for detection of dolphin clicks. In spite of this, a False Positive rate higher than expected was revealed, especially during 2005. Consequently, a post-processing analysis was focused on the files categorized as FP to understand in which conditions the algorithm detected clicks when human operators did not. Four possible indicators were analysed for each file: number of clicks detected, mean amplitude of clicks, mean peak in frequency of clicks and presence of dolphin whistles. Median number of clicks detected per file was lower than in the other categories of files, with 25 pulses in the 2005 dataset (Percentiles, 25^th^: 12; 75^th^: 51; 95^th^: 267) and 16 pulses in the 2006 dataset (Percentiles, 25^th^: 10; 75^th^: 30; 95^th^: 97) (Fig. 2b). Both amplitude and frequency peak of the selected clicks were lower than the other categories (Fig. 2b). These results allowed us to assume that in these cases the animals were more distant from the station. Moreover, whistles emitted by dolphins were present in 71.06% of FP recordings in 2005 and in 61.53% of FP recordings in 2006, by looking at the results of spectrogram analysis by human-operators (manual analysis).

### Temporal patterns in dolphin vocalizations

**Figure 3:**
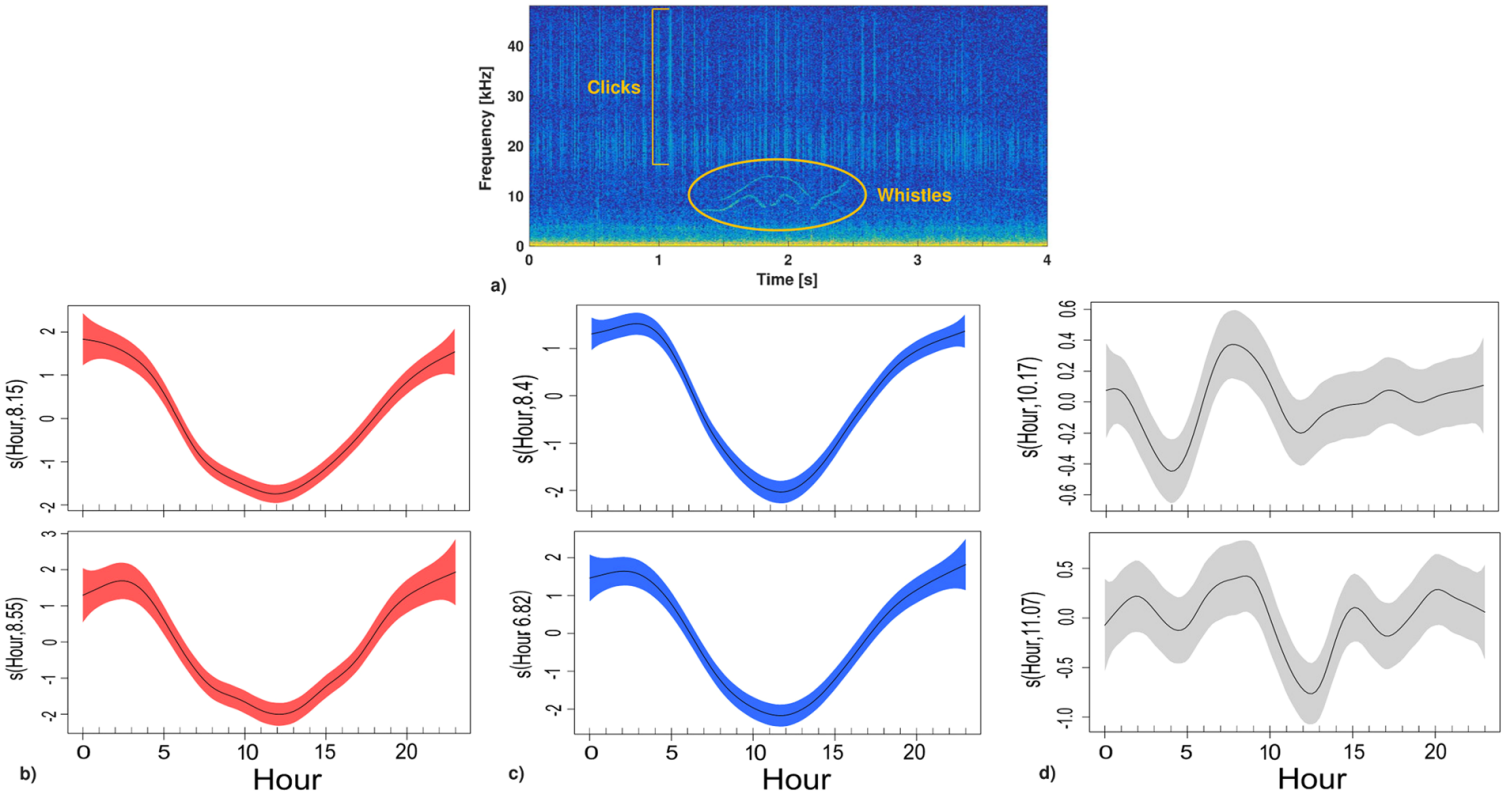
Daily pattern. (**a**) Spectrogram (nfft = 2048; overlap = 50; Hann window) of dolphin vocalizations investigated: clicks and whistles. (**b**) Click probability of presence for the automatic analysis (in red) as a function of hour of day (GAM model, 2005: N = 3736, R^2^ = 0.215, Deviance explained = 19.7%, Hour: χ^2^ = 619.7, P < 0.001; 2006: N = 1758, R^2^ = 0.266, Deviance explained = 24.1%, Hour: χ^2^ = 349.3, P < 0.001). (**c**) Click probability of presence for the manual analysis (in blue) as a function of hour of day (GAM model, 2005: N = 3736, R^2^ = 0.297, Deviance explained = 23.2%, Hour: χ^2^ = 914.3, P < 0.001; 2006: N = 1758, R^2^ = 0.337, Deviance explained = 27.7%, Hour: χ^2^ = 459, P < 0.001). (**c**) Whistle probability of presence for the manual analysis (in grey) as a function of hour of day (GAM model, 2005: N = 3736, R^2^ = 0.0084, Deviance explained = 0.883%, Hour: χ^2^ = 35, P < 0.001; 2006: N = 1758, R^2^ = 0.0194, Deviance explained = 2.1%, Hour: χ^2^ = 37.53, P < 0.001). (**b–d**), (*top*) 2005; (*bottom*) 2006; (*shaded areas*) 95% confidence intervals.

Daily temporal patterns of acoustic presence or absence of dolphin vocalizations (clicks and whistles, Fig. 3a) were analysed using Generalized Additive Models (GAMs). Detection of echolocation clicks, both with manual and automatic methods, showed an evident diel cycle, with higher biosonar activity of dolphins during the nightly phase (Fig. 3b,c). Hour of the day is shown by the model as a relevant predictor for dolphin clicks occurrence. The number of files with echolocation signals was higher during nighttime, both in 2005 and 2006. In contrast, whistles (communication sounds) did not show a similar daily pattern such as the clicks (Fig. 3d).

**Figure 4:**
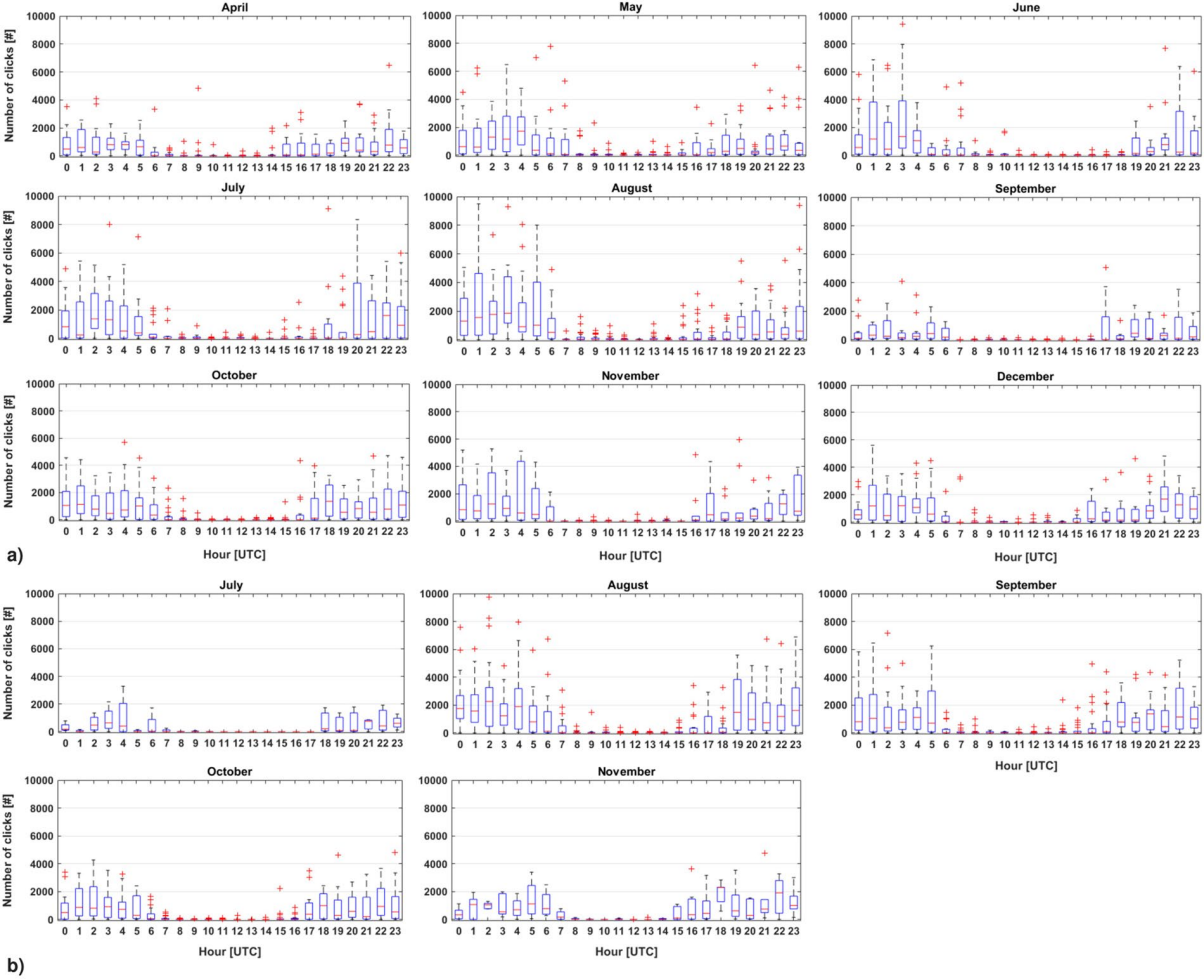
Boxplot of the number of clicks recorded per hour in each month. In each box, the central mark indicates the median, and the bottom and top edges of the box indicate the 25^th^ and 75^th^ percentiles, respectively. Whiskers extend to the most extreme data points not considered outliers, and outliers are plotted individually using the ‘+’ symbol (in red). (**a**) Data acquired in 2005; (**b**) Data acquired in 2006.

**Figure 5:**
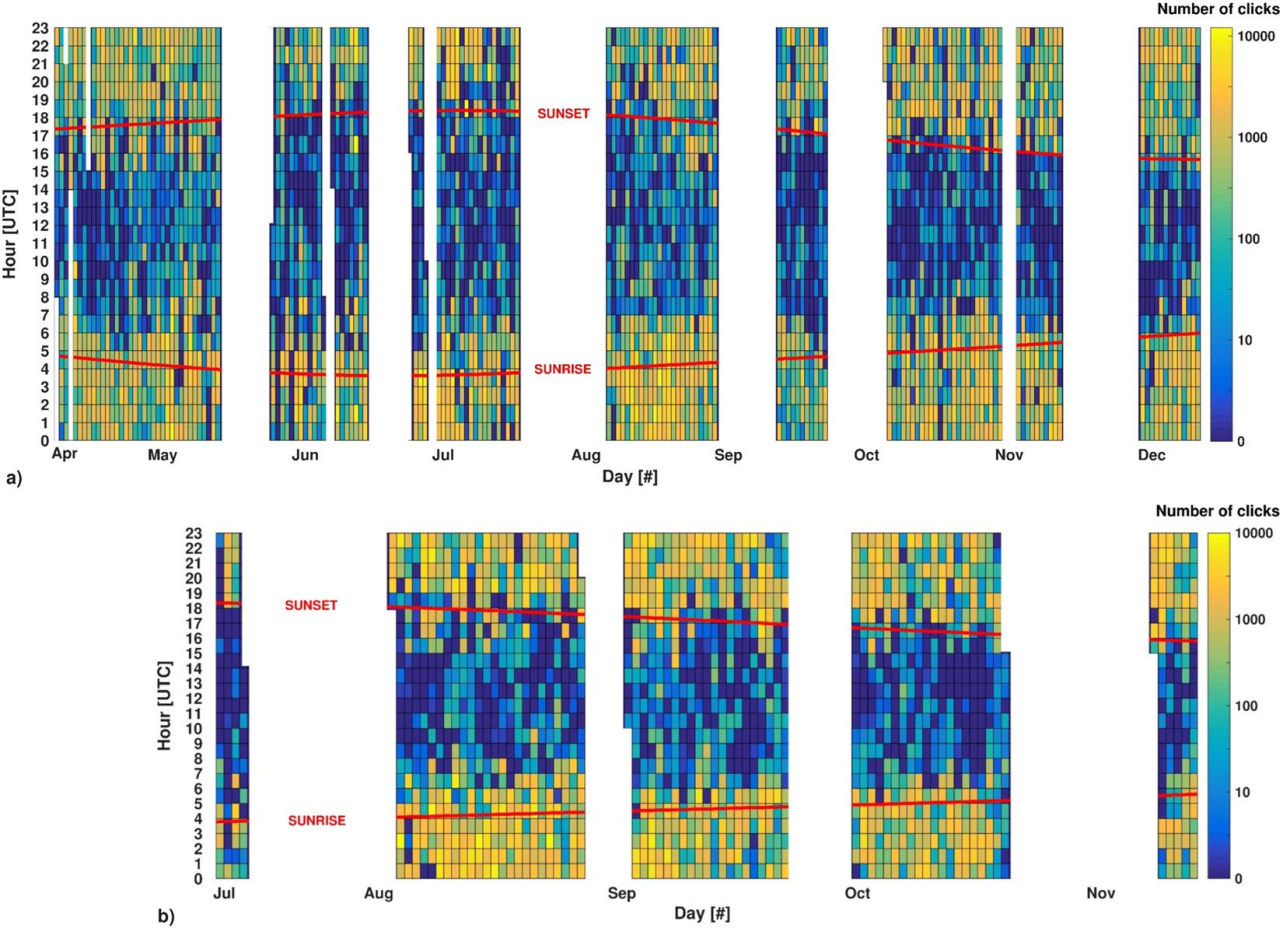
Influence of sunrise and sunset on biosonar activity of dolphins. Red lines indicate variation of sunrise and sunset timing through the months. (**a**) Number of clicks detected per hour during 2005; (**b**) Number of clicks detected per hour during 2006.

Results of the algorithm showed that number of recorded clicks per file increased during night hours for all months, with the same daily trend (Fig. 4). During the two-year survey, the highest number of clicks was recorded in the period immediately after sunset until the first hours of sunrise, following the daylight seasonal variation. Timing of the diel phenomenon was linked to variation of the hours of lightness/darkness during different months (Fig. 5).

**Figure 6:**
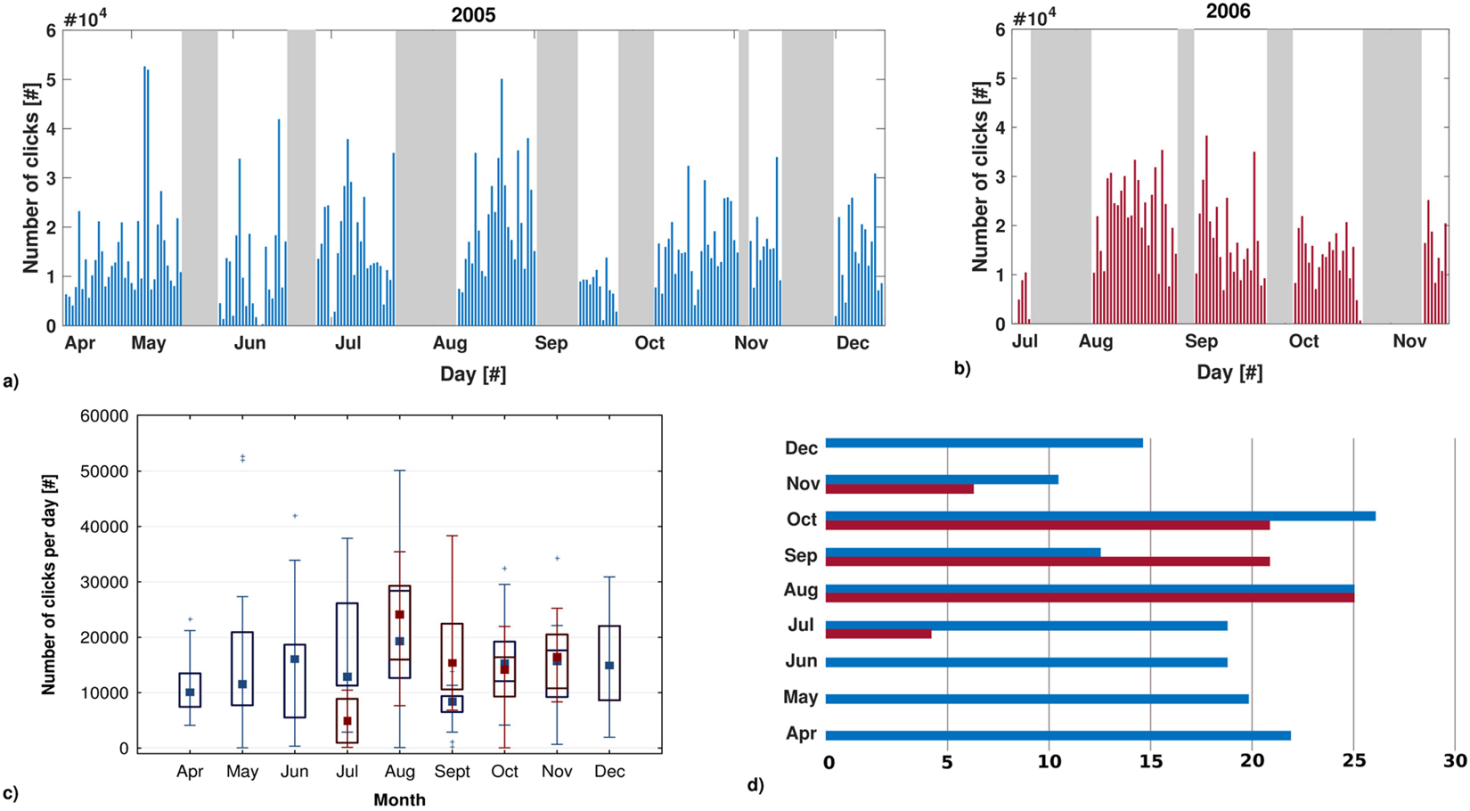
Number of clicks recorded per day (5 min/h) (**a**, **b**, ﻿**c**). (**a**) 2005, from 9^th^ April to 15^th^ December (3,736 files); (**b**) 2006, from 11^th^ July to 14^th^ November (1,758 files); days in which the OνDE station did not acquire data are showed in grey. (**c**) In each box (blue: 2005; red: 2006), the central mark indicates the median, and the bottom and top edges of the box indicate 25^th^ and 75^th^ percentiles, respectively. Whiskers extend to the most extreme data points not considered outliers, and outliers are plotted individually using the ‘+’ symbol. (**d**) Acquisition effort in number of days of recordings per month over the two years.

Number of clicks detected per day differed significantly across months (Kruskal-Wallis nonparametric test for 2005: H[8,N = 161] = 25.44, p = 0.0013; for 2006: H[4,N = 79] = 22.73, p = 0.0001). Statistical differences were identified for 2005 between Jul and Sept, Jul and Aug, Sept and Oct and for 2006 between Jul and Aug, Aug and Oct (p < 0.05, Multiple Comparisons test 2-tailed). The peak of dolphin biosonar activity was recorded in August during both years of monitoring (Fig. 6).

### Influence of the lunar cycle

**Figure 7:**
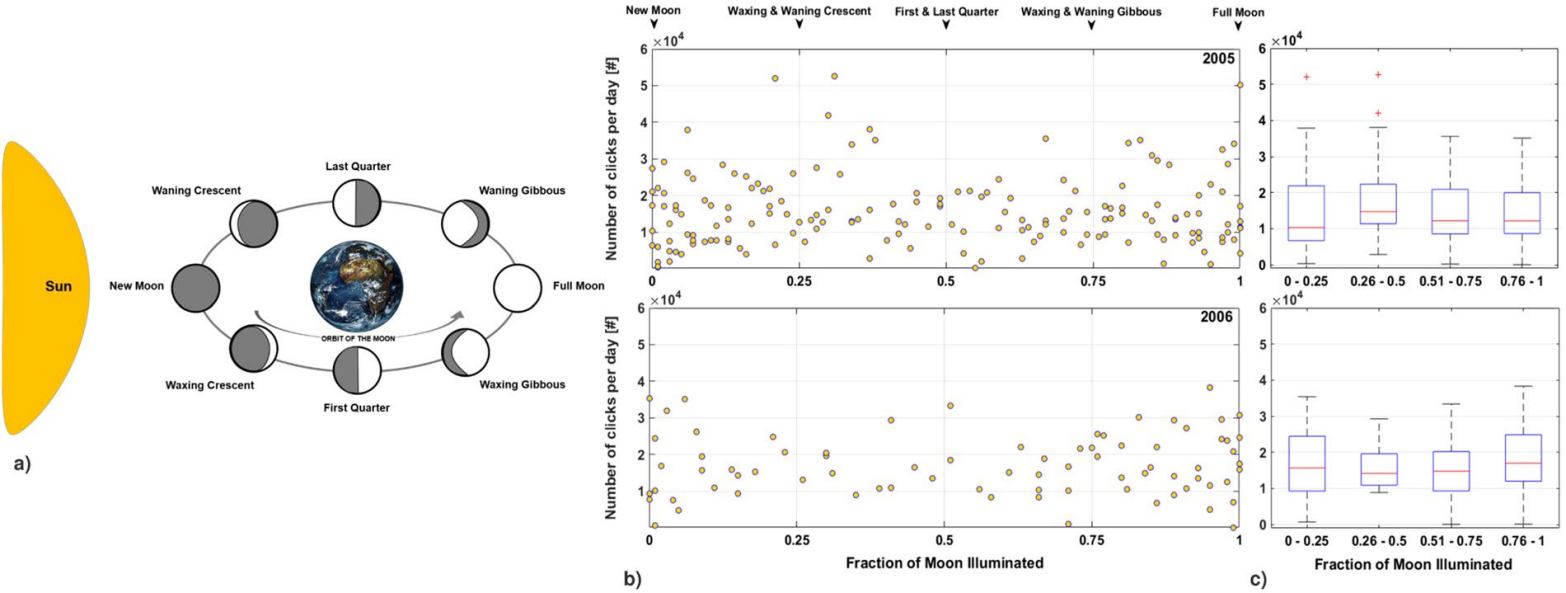
Influence of the Lunar Cycle. (**a**) Variation of the fraction of moonlight in relation to different lunar phases. (**b**) Scatterplot shows the number of clicks recorded per day (5 min/h) in relation to the increase of the fraction of Moon illuminated (top: 2005; bottom: 2006). (**c**) Number of clicks recorded per day with the dataset divided into four categories of moon illumination. On each box, the central mark indicates the median, and bottom and top edges of the box indicate 25^th^ and 75^th^ percentiles, respectively. Whiskers extend to the most extreme data points not considered outliers, and outliers are plotted individually using the ‘+’ symbol (in red).

The Moon’s brightness was investigated as a potential environmental parameter affecting dolphin biosonar activity (Fig. 7a). Number of clicks recorded per day was not influenced by the fraction of moon illuminated in relation to the daily lunar phase (Fig. 7b). Considering four levels of brightness (ratio of moon illuminated, from 0: New moon to 1: Full moon), results showed that lunar cycle did not influence dolphin biosonar in the deep pelagic waters of the OνDE site during the two-year survey (Fig. 7c). A multiple comparisons test (2-tailed) and Kruskal-Wallis non-parametric test did not show differences in click numbers between the four categories of brightness (2005 test: H[3,N = 170] = 3.02, p = 0.3876; 2006 test: H[3,N = 79] = 2.23, p = 0.5245).

### Detection range of the OνDE station

**Figure 8:**
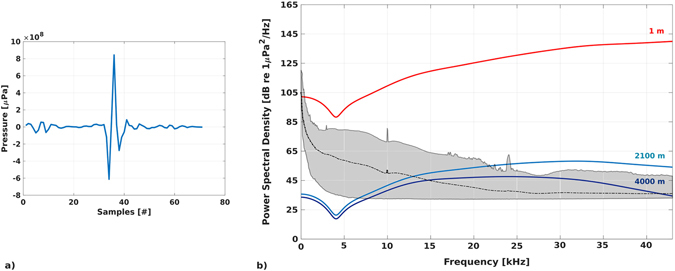
Detection range of the OνDE station. (**a**) Waveform of a click emitted by a striped dolphin (*Stenella coeruleoalba*) recorded (fs: 300 kHz) during a boat-based acoustic survey conducted in the Central Mediterranean Sea. (**b**) Transmission Loss (TL) model of the click to recognize the variation of Power Spectral Density (PSD, dB re 1 µPa/√Hz) during signal propagation. In red, PSD of the signal as source spectral density; in light blue, PSD of the click after 2,100 m of propagation; in blue, PSD of the click after 4,000 m of propagation. The grey area represents upper and lower limits of average PSDs calculated for the OνDE dataset recorded using channel H3. The black curve is the average calculated over all PSDs.

Transmission Loss (TL) of a click emitted by striped dolphin (*Stenella coeruleoalba*), the most abundant pelagic dolphin in the Mediterranean^38^, was evaluated to estimate the detection range of the OνDE station. According to the developed TL model of the click and to the average PSD calculated on all dataset, the OνDE station - with sensors installed at 2,100 m of depth – detected dolphin clicks in a range of about 4 km (Fig. 8).

## Discussion

Passive acoustic monitoring is rapidly growing as a relatively low-cost, high-resolution sampling approach for gaining novel information on marine animals^2^. This study presents a detailed comparison between two detection methods, showing that the human-operated analysis was improved using the automatic algorithm. These complimentary approaches provided new ecological information, and a method to rapidly analyse large acoustic datasets for dolphin activity. The OνDE deep-sea cabled station allowed a long-term monitoring program of the acoustic sources present in a wide deep area in the Gulf of Catania (Western Ionian Sea). For the first time, data acquired in deep-pelagic waters, at a depth of 2,100 m, allowed us to reveal multiple scales of temporal variation in the biosonar activity of these dolphin populations.

Differences in the detectability of dolphin clicks were associated with the application of deep-sea monitoring and the acoustic features of the ultrasonic signals investigated. The recordings marked as False Positive presented number, amplitude and peak frequency of clicks lower than other categories (the animals were probably at greater distance). In the majority of FP recordings, the acoustic presence of whistles emitted by dolphins was reported in both years. Furthermore, OνDE sensors recorded a mean peak frequency of 23 kHz for clicks detected during the two-year monitoring, a value much lower than that at the source^3^. The results led us to hypothesize that the algorithm had greater accuracy for the detection of single clicks, considering the high frequency components of the signal and the depth of the sensors (2,100 m). Species identification of the dolphins recorded by the OνDE station was not possible due to the sampling frequency (fs = 96 kHz) and the depth of the sensors used. Even though these conditions could not be met in this work, relevant information on the acoustic behaviour and ecology of dolphins in general was obtained by studying their biosonar activity via the long-term PAM survey. While the automatic selection of clicks emitted by sperm whales or beaked whales is relatively simple using time and frequency parameters, the species-specific differences have not been clearly documented for all species of dolphins^18–20^. A reliable species classifier for wild dolphins should take into account the detailed orientation of all recorded clicks and their acoustic spectrum (with large bandwidth recording)^18^. Incorporating these considerations into future species-specific echolocation detectors will allow for a more comprehensive analysis of delphinid community composition and drivers of biosonar detection variability.

A variety of Odontocete species have been observed worldwide to be more acoustically active at night, especially during short-term, shallow water and/or coastal monitoring^40–46^. Our results show that animals were detected both during day and night hours but clicks are used primarily during nighttime. The diel trend found in number of clicks detected likely reflects changes in the acoustic behaviour of dolphins in relation to environmental stimuli, namely the absence of light. This confirms that echolocating activity occurs mostly under the cover of darkness, when visual information is unavailable. Conversely, patterns in non-biosonar vocalizations (whistles) indicate that the need to maintain social contact through whistles is more constant and unrelated to different light levels. This suggests that the diel change in biosonar activity detected is not linked to a periodic daily movement outside the detection range of OνDE, but to a real change in vocalization rhythms. The absence of clicks during daylight indicates that echolocation was not the primary sense used for environmental recognition during daylight hours, but is relied upon at nighttime. The high number of echolocation clicks recorded during the night is also likely related to the dynamics of the trophic chain in the epi-mesopelagic layers of the open sea (from surface to 1000 meters). During the nocturnal migration of pelagic plankton, fish and cephalopods migrate towards the surface after sunset, with a consequent increase in cetacean feeding activity within the epi-mesopelagic zones^10, 40^. Therefore, the echolocation activity of dolphins is consistently connected to the hours of darkness in a pelagic environment, corresponding to these feeding conditions.

The daily cycle of dolphin biosonar activity in the detection area was consistent across sampling years and during every month. The highest number of clicks detected per day was recorded in August both in 2005 and in 2006. This month-to-month variation in the amount of echolocation signals is likely an indication of seasonal changes in the abundance and distribution of delphinids, possibly related to prey distribution and foraging behaviour as has been shown for other odontocetes^1^. During experiments on a seasonal basis, Madurell *et al*.^47, 48^ studied the spatial and temporal structure of fish assemblages and the daily food consumption of dominant demersal species (e.g. sharks and fishes) in a bathyal area of the Eastern Ionian Sea. They found that fish biomass was maximal in August. During this month, a dominance of pelagic feeders coincided with a higher biomass of benthopelagic zooplankton taxa preyed by fish, with significant ecological connections between trophic levels^47^. In this scenario, and if ecological dynamics are the same in the bathyal area of the Western Ionian Sea, the highest biosonar activity of dolphins (pelagic feeders) in August could be related to increased prey availability. While August is a part of the “boreal summer” at the OνDE latitude, when darkness is minor, the trophic hypothesis could explain why it was the month with the highest average daily number of clicks. Lunar cycle did not have a significant influence on detections of dolphin biosonar in deep pelagic waters monitored here. This is in contrast with other studies, which showed correlations between dolphin vocalizations and lunar phase^44–46, 49^. This difference could relate to lunar phase having a greater influence on dolphin ecology in tidally-influenced coastal environments compared to further offshore, and to the relatively low tidal amplitudes in the Mediterranean Sea compared to the oceans. It is important also to consider that this study measured echolocation patterns for multiple species of delphinids in combination, and future species-specific analyses are needed to test hypotheses about how seasonal patterns and biogeographical variation influence any one species. These results show the complexity of relationships between environmental drivers and marine mammal habitat use and behaviour across geographical gradients, and further highlight the utility of acoustic monitoring to elucidate spatiotemporal variation in difficult to observe marine mammals.

## Conclusions

Determining spatial and temporal patterns in marine mammal vocalizations is key to understanding their behaviour and ecology in the natural environment. In the deep pelagic waters of the OνDE station, the use of an algorithm to automatically detect dolphin clicks was a reliable method to investigate echolocation activity during a two-year PAM survey, reducing time requirements of data analysis and improving the human-operated analysis with new ecological and ethological information. An evident diel pattern in dolphin biosonar activity was revealed. This temporal dynamic occurred in all months, with the same daily trend in 2005 and in 2006, starting immediately after sunset and ending during the first hours of sunrise. The click distribution reflects changes in the behaviour of dolphins related to environmental and trophic stimuli. The dominance of clicks during hours of darkness confirms that echolocation is a primary sensory mode used by dolphins for environmental recognition during night, and that cetacean feeding increases at this time of day, corresponding to the nocturnal migration of pelagic plankton, fish and cephalopods toward the surface. A seasonal increase of fish biomass and changes in dolphin abundance in the Ionian Sea could also explain the higher biosonar activity recorded in August during both 2005 and 2006.

The deep sea acoustic monitoring program provided detailed evidence of the acoustic behaviour of delphinids over two years, but high sample rate recordings (>200 kHz) are needed to ultimately elucidate spectral differences and differentiate among the various species present. On-site observations, new tools and analyses (e.g. tagging, photo-id, ecotoxicology, genetics, etc.) will be needed to further investigate the health and conservation status of the dolphin species present in the area and to match the information received by PAM activity through the sea-floor cabled station. The KM3NeT Collaboration and the EMSO Network are currently working on the development and operation of new fixed deep-sea observatories for the real-time and long-term monitoring of the Southwestern Ionian Sea. The acquisition of new acoustic data is ongoing and will allow us to continue to apply and extend our algorithm to further investigate the occurrence and vocal activity of different marine organisms in the area. The study of the sound components recorded will improve our knowledge of the deep soundscape of the Mediterranean Sea and the ecology of pelagic marine organisms.

## Methods

### Large dataset acquisition

The OνDE acoustic dataset was acquired using the software *WaveinRecorder* (CIBRA)^50^. The number of recordings acquired changed month-to-month over the years, owing to system maintenance or technical reasons. The first 5 minutes of each hour were recorded. The dataset analysed was acquired in 2005, from 9^th^ April to 15^th^ December (3,736 files), and in 2006, from 11^th^ July to 14^th^ November (1,758 files), and consisted of 5,494 recordings.

### The Ocean noise Detection Experiment

The OνDE station was in operation in the INFN Test Site from January 2005 to November 2006. The Test Site consists of a laboratory located in the Port of Catania (Sicily, Italy), a 28 km long electro-optical submarine cable laid on the seafloor, and two terminations anchored at a depth of 2,100 m, 25 km from the shore (Gulf of Catania, Western Ionian Sea). OνDE was installed on the mechanical frame hosting the connectors of the South Cable Termination Frame, located at Latitude 37°32.681′N, Longitude 15°23.773′E (Fig. 1a)^31, 51^. This experiment was the first long-term scientific installation for monitoring in real time the acoustic environment in a deep area of the Mediterranean Sea^24^. The OνDE acoustic antenna was made of four piezoelectric omnidirectional hydrophones (RESON TC4042-C), arranged in a tetrahedral shape (Fig. 1b). The hydrophones were certified by RESON^52^ to operate at 2,500 m depth with a mean receiving sensitivity of −195 ± 3 dB re V/μPa, linear over a wide range of frequencies: from few tens Hz to about 50 kHz (coupled to a 20 dB preamplifier made by RESON). Hydrophone data were sampled underwater using a pair of stereo A/D converters from Cirrus Logic^53^. A sampling frequency of 96 kHz with 24-bit quantization was used; the input voltage range of the ADCs was 4 V_pp_. The two stereo streams were kept synchronized by a common clock. Two electro-optical modems provided the transmission of the digital audio streams through the submarine electro-optical cable. On shore, two soundboards (RME, DIGI96/8-PAD) were used to acquire in real-time the data streams 24/24 h. The software *WaveinRecorder* (CIBRA)^50^ was interfaced to the hardware devices for the data acquisition and to keep the two audio streams synchronised. Due to storage size limitations, the first five minutes of each hour were stored; this produced twenty-four samples per day, each file containing the four audio channels in 32-bit integer format (size of 450 MB per file). The data channel analysed in this work was from the hydrophone installed on the top of the frame (Fig. 1, H3), placed at about 3.5 metres above the seafloor.

### Manual Analysis: *SeaPro software*

A manual survey of the data provided information on the acoustic presence of biological sounds. Three operators - with experience in PAM - detected the species belonging to the Family *Delphinidae* through spectrogram visualization and listening analysis, based on known descriptions of marine mammal vocalization features (e.g., time-frequency, spectral content, intensity). To confirm the occurrence of dolphin vocal emissions, *SeaPro* software^50^ was used to play the recordings and to produce a synchronous real-time and high-resolution spectrographic display. The systematic sampling protocol was designed to minimize operator-influenced bias. Operators were trained to have homogeneous discrimination ability and uncertain sounds were checked by the team. The measurements produced by the manual analysis that were used in this work were: (1) Presence/Absence in each recording of Clicks emitted by Family  *Delphinidae*, and (2) Presence/Absence in each recording of Whistles emitted by Family *Delphinidae*.

### Automatic Analysis: algorithm for click detection

An algorithm was developed in MATLAB to analyse the acoustic data acquired by the OνDE station. This was an updated version of the script used by Buscaino *et al*.^54^ to describe acoustic behaviour of the bottlenose dolphin (*Tursiops truncatus*) in the Central Mediterranean Sea. Dolphin clicks were automatically detected in five main steps:

1. Signal conditioning process applied to all data (15 kHz high-pass filter). The data were first filtered with a 15 kHz high-pass filter (Butterworth, 4th order, zero-phase digital filtering). This filter allowed us to remove the majority of the pulsed signals generated by sperm whales and ships (first peak frequency and centroid frequency usually lower than 15 kHz).
2. Application of the Teager-Kaiser Energy Operator (TKEO). An energy detector based on the Teager-Kaiser Operator was used to identify the dolphin clicks^18, 20, 54–57^. The TKEO responds to the increase of energy due to an instantaneous signal characterized by high frequency.
3. Automatic calculation of the threshold and extraction of “candidate clicks”. The adaptive threshold was applied using an iterative process within each 5-minute recording. Starting from a first threshold equal to the average value of the signal evaluated by the TKEO, the algorithm analysed the variation of the number of samples triggered during a sequential raise of the threshold. The applied technique allowed us to move the threshold in relation to the change of the background noise from one file to the next. The application allowed the extraction of each candidate click in a time window of 128 samples (1.3 ms, centred on the detected peak).
4. Signal conditioning applied to the original waveform of the candidate clicks (3 kHz high-pass filter), oversampling and measurement of their principal acoustic features. The original waveform of the time window selected was filtered with a 3 kHz high-pass filter (Butterworth, 4^th^ order). This time window was resampled at a sampling frequency eight times higher and the following acoustic features were measured:
  Pulse Duration [ms]: the duration of the pulse was determined from the envelope of the TKEO, identifying the pulse peak. The onset and termination of the click signal were defined as the points at which 10% of the peak value was reached.
  Peak to Peak Amplitude (Vpk-pk) [V]: the difference between the maximum positive and the maximum negative amplitudes of the waveform.
  Frequency Peaks (fp) [kHz]: 1st, 2nd, and 3rd peak frequencies are the frequencies corresponding to the highest, second, and third amplitude, respectively, determined for the Power Spectral Density.
  Number of Peaks in Frequency (No. f_p_) [#]
  Centroid frequency (fc) [kHz]: the frequency value that divides the spectrum in halves of equal energy^14^.
  Bandwidth_RMS_ [Spectral Standard Deviation around Centroid Frequency]: bandwidth RMS (Root Mean Square) is a measure of the spectral standard deviation around the centroid frequency of the spectrum^14^.
  Q_RMS_ [Centroid Frequency/Bandwidth_RMS_]
  Number of Zero Crossing [#]: number of times the signal crosses zero.
5. Selection of dolphin clicks using known parameters of the signal. We measured the main features of the signals detected for the characterization of echolocation clicks emitted by dolphins. In each file, the acoustic presence of dolphin biosonar was confirmed when at least five events were detected. The signal was considered a dolphin click according to the following conditions:

- Pulse Duration < 0.4 ms
- Vpk-pk < 4 ⋅ 0.05
- No. f_p_ < 4
- f_p_ + f_c_ > 30 kHz
- Q_RMS_ > 1
- 2 < Zero Crossing < 20

### Biosonar detector performance

A confusion matrix was generated to check the automatic detection of dolphin clicks by the developed algorithm, compared to human-operated analysis. The confusion matrix is a specific analysis that allows visualization of the performance of a detection algorithm^58^. In this work, the manual analysis represented the instances of *True condition* (columns), while the automatic analysis was considered the *Predicted class* (rows). Therefore, the recordings were classified in four different categories, in relation to the presence of dolphin clicks by means of the two methods (True Positive-TP, True Negative-TN, False Positive-FP, and False Negative-FN).

### Temporal patterns

Generalized Additive Models (GAMs)^59^ were used to evaluate the temporal patterns of dolphin click occurrence. A binomial-based GAM, using a logit link function, was performed in relation to the presence/absence of clicks. We tested hour of the day as a continuous explanatory variable. GAMs were fitted with the MGCV library^60^ in R^61^, and the splines library was used to build cubic regression splines^59^. Each spline had 24 internal knots corresponding to the different hours.

Sunset and sunrise times and information on the lunar phase at the site of the OνDE station were obtained from the website of the US Naval Observatory (http://aa.usno.navy.mil). We considered four ranges of moon illumination - in relation to the lunar phases - to examine if the acoustic behaviour of dolphins changed in relation to the lunar cycle. Four levels corresponding to the ratio of moon illuminated were applied: lev.1 = 0.00–0.25; lev.2 = 0.26–0.50; lev.3 = 0.51–0.75; lev.4 = 0.76–1.00.

### Detection range of the OνDE station

The Transmission Loss (TL) of a click emitted by striped dolphin (*Stenella coeruleoalba*), the most abundant pelagic dolphin in the Mediterranean^38^, was calculated to evaluate the extent of the detection range of the OνDE station. Acoustic data on this species were collected during boat-based surveys conducted in the Central Mediterranean Sea^54, 62^, with the animals very close to the boat. The *source spectral density level* (dB re 1µPa/√Hz @ 1 m) of the most intensive click was estimated. Then, considering the emission of the click by a dolphin close to the surface, we applied a Transmission Loss (TL) model for all frequencies to recognize the variation of the Power Spectral Density (PSD, dB re 1µPa/√Hz) during signal propagation. We considered attenuation due to geometric spreading and absorption processes related to seawater properties^63, 64^. The geometric spreading model measured spherical spreading when distance from the source (R) was lower than the depth of the OνDE station (D = 2,100 m), and cylindrical spreading for higher distances.

For spherical spreading, we used the formula:

$${\rm{TL}}=20\,\mathrm{log}\,{\rm{R}}+{\rm{\alpha }}R$$

and for cylindrical spreading, the formula:

$${\rm{TL}}=10\,\mathrm{log}({\rm{D}}* {\rm{L}})+{\rm{\alpha }}R$$

where L is the distance from the projection point on the surface of the OνDE station and α is the absorption coefficient. The α coefficient takes into account the contribution of temperature, hydrostatic pressure, and salinity^65^. The CTD (Conductivity, Temperature and Depth) data used to determine α were acquired during previous campaigns in the Gulf of Catania, using an MK-317 CTD from Idronaut^66^. The detection range of the OνDE station (2,100 meters of depth) for dolphin biosonar was estimated considering the PSD curve of the click that matches the average PSDs calculated for the entire dataset^24^ at 15 kHz (first filter used for click detection).
